# The complex metabolic interactions of liver tissue and hepatic exosome in PCOS mice at young and middle age

**DOI:** 10.3389/fphys.2022.990987

**Published:** 2022-09-20

**Authors:** ShanHu Gao, Fei Long, Zheng Jiang, Jun Shi, DongXue Ma, Yang Yang, Jin Bai, Ting-Li Han

**Affiliations:** ^1^ State Key Laboratory of Ultrasound in Medicine and Engineering, College of Biomedical Engineering, Chongqing Medical University, Chongqing, China; ^2^ Jockey Club School of Public Health and Primary Care, The Chinese University of Hong Kong, Shatin, China; ^3^ Department of Obstetrics, The First Affiliated Hospital of Chongqing Medical University, Chongqing, China; ^4^ Department of Obstetrics and Gynaecology, The Second Affiliated Hospital of Chongqing Medical University, Chongqing, China

**Keywords:** PCOS (polycystic ovarian syndrome), IR (insulin resistance), liver, exosome, metabolomics, aging

## Abstract

Polycystic ovary syndrome (PCOS) is a common age-related endocrinopathy that promotes the metabolic disorder of the liver. Growing evidence suggests that the pathophysiology of this disorder is closely associated with the interaction between the liver and its exosome. However, the underlying mechanism of the interactions remains unclear. In this study, we aimed to investigate the metabolite profiles of liver tissues and hepatic exosomes between normal (*n* = 11) and PCOS (*n* = 13) mice of young- and middle-age using gas chromatograph-mass spectrometry (GC-MS) based metabolomics analysis. Within the 145 identified metabolites, 7 and 48 metabolites were statistically different (*p* < 0.05, *q* < 0.05) in the liver tissue and exosomes, respectively, between PCOS and normal groups. The greater disparity in exosome indicated its potential to reflect the metabolic status of the liver. Based on hepatic exosome metabolome, the downregulations of glycolysis and TCA cycle were related to hepatic pathophysiology of PCOS independent of age. Fatty acids were the preferred substrates in young-age-PCOS liver while amino acids were the main substrates in middle-age-PCOS liver for the processes of gluconeogenesis. Overall, this study enables us to better understand the metabolic status of the PCOS liver at different ages, and exosome metabolomics shows its potential to gain the metabolic insights of parental cell or source organ.

## Introduction

Polycystic ovary syndrome (PCOS) is a prevalent reproductive disorder among female population. The epidemiological studies found that the prevalence of PCOS ranges from 10% to 15% worldwide (Azziz et al., 2016; MacParland et al., 2018; Abbott and Dumesic, 2021). Most of the patients with PCOS have features of metabolic abnormalities such as dyslipidemia, hyperandrogenism, insulin resistance, obesity, and hyperinsulinism, that result in the increased risk of diabetes mellitus, hypertension, depression, endometrial cancer, cardiovascular diseases etc. (Azziz, 2018; Liu et al., 2020). Liver is the largest metabolic organ that provides immune and metabolic functions. Liver functions are also affected by PCOS. In recent years, studies found that non-alcoholic fatty liver disease (NAFLD) was more prevalent in PCOS groups (50.6%) than in other normal groups (34.0%), illustrating that there is probably existing an interplay or shared aetiology between PCOS and liver dysfunction (Vassilatou, 2014; Macut et al., 2016; Targher et al., 2016; Anagnostis et al., 2018; Salva-Pastor et al., 2019; Sarkar et al., 2020).

Extracellular vesicles (EVs) are lipid-bilayer-structure microparticles secreted by almost all cell types. Exosomes are EVs with a diameter range of 50–150 nm, which origins from the cellular endocytic system (van Niel et al., 2018). As a carrier in the intercellular communication process, exosome can deliver proteins, DNA, RNA, and metabolites. It plays important roles in molecular transport, membrane formation, stress response, and other biological functions of cells (Wortzel et al., 2019). Studies found that the intracellular traffic of exosome’s cargo depended not only on the endocytic pathway but also on the biosynthetic secretory pathway for their release, suggesting that the contents in exosomes could reflect the pathological and physiological states of parental cells or source organs (Mashouri et al., 2019; Teruel-Montoya et al., 2019; Reclusa et al., 2020; Xia et al., 2020). In fact, several studies showed that liver-derived exosomes could be used to reflect the pathophysiology of liver diseases (Masyuk et al., 2013; Sung et al., 2018; Wang et al., 2019), which means such a method can probably be successfully applied in this study to discover the underlying hepatic metabolic dysfunction in the PCOS liver.

Metabolomics is the comprehensive profiling of small molecule metabolites in biofluids, cells, or tissues which has evolved rapidly in these years. Due to the high sensitivity of metabolites to external stimuli and internal signaling, metabolomics can provide more intuitive and more efficient biological phenotypes of human subjects or animal models compared to other “-omics” (Johnson et al., 2016; Liu et al., 2017; Newgard, 2017; Wishart, 2019). Since PCOS is a metabolic disorder, metabolomics would be useful in studying the pathophysiology of PCOS. In recent years, some metabolomic analyses of PCOS have been performed, and the results showed that the abnormalities in PCOS are mainly associated with the metabolism of lipids, fatty acids, sphingolipids, glycerophospholipids, carbohydrates and amino acids. Sample type in these studies includes blood, urine, and follicular fluid (Zhao et al., 2012; Daan et al., 2016; Rajska et al., 2020). However, the metabolome of body fluid only represents the general status instead of the specific organ system of the whole body. Owing to the source heterogeneity of exosomes, we are able to detect the metabolic change in specific organs through analyzing specific organ-derived exosome contents. Considering the roles of exosomes, the metabolic abnormalities in PCOS can potentially cause changes to the qualitative and quantitative profile of the metabolites in hepatic exosomes, which can be measured by mass spectrometry-based metabolomics.

In this study, we described the alterations in liver tissue and hepatic exosomes metabolome in PCOS mice as compared to the controls and then performed pathway analysis to investigate the potential hepatic pathogenesis in PCOS mice. Additionally, the role of aging in this metabolic pattern was also investigated.

## Materials and methods

### Generation of Polycystic ovary syndrome mouse models

**FIGURE 1:**
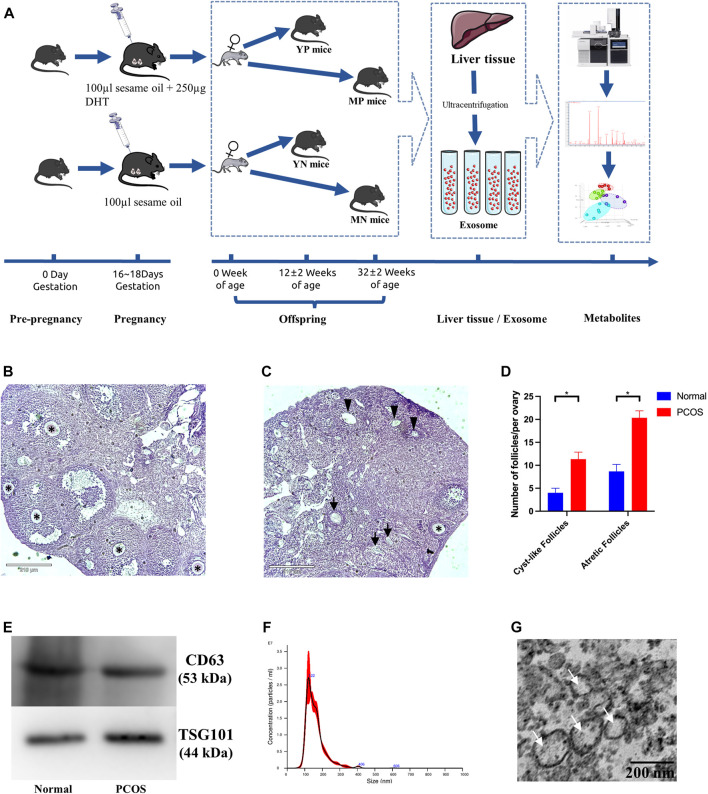
**(A)** Overview of PCOS modeling process. Morphological appearance and evaluation of follicles in affected ovaries from **(B)** normal (left) and **(C)** PCOS mice. The normal follicles were indicated with black asterisks (*), the atretic follicles were indicated with black arrows (↑), and the cystic follicles were indicated with black triangles (▲). **(D)** Bar graphs show the number of atretic follicles and cystic follicles in PCOS and normal mice (*Student’s *t*-test, *p* <0.05). **(E)** Western blotting validated exosomes through CD63 and TSG101. **(F)** The size distribution of exosomes measured by the method of NTA. **(G)** Exosomes examined by Transmission electron microscopy (TEM).

A total of 10 clean inbred C57/BL6 female mice, 6–8 weeks old were purchased from the Experimental Animal Center of Chongqing Medical University. The PCOS models were established based on the protocol of prenatally androgenized (PNG) (Caldwell et al., 2014). Firstly, female mice were paired with male mice and mated overnight, and the day of formation of a vaginal plug was taken as day 1 of gestation. On days 16–18 of gestation, five pregnant female mice were injected with 100 µl sesame oil (normal group), while another five pregnant mice were injected with 100 µl sesame oil containing 250 µg dihydrotestosterone (DHT, Sigma, United States) (PCOS group). The female offsprings were grouped according to age (12 ± 2 weeks as the young-age group, 32 ± 2 weeks as the middle-age group) and sacrificed by cervical dislocation. A total of six ovaries (PCOS: *n* = 3, Normal: *n* = 3) were fixed with 4% paraformaldehyde, immobilized in paraffin, sectioned (5 μm) and stained with hematoxylin and eosin (H&E). The numbers of normal follicles, atretic follicles and cystic follicles were counted throughout each ovary using an Inverted microscope (Revolve, United States). Plasma levels of testosterone (T) were measured by Enzyme-Linked Immunosorbent Assay (ELISA, Jing Mei, China). Overview of modeling process is shown in Figure 1A. The use of experimental procedures involving animals were approved by the ethics committee of Chongqing Medical University. All procedures involving animals were conducted with the guidelines of the Institutional Animal Care and Use Committee (IACUC) of Chongqing Medical University.

### Digestion of liver tissue and isolation of exosome

100 mg type IV collagenase (Biosharp, China) was added into 100 ml Hanks Balanced Salt Solution (HBSS, Beyotime, China) as a digestion solution. The portal vein was ligated, and cannulation was performed 5–10 mm below the ligature. The liver was infused with HBSS (at the flow rate of 1–2 ml/min) until the tissue turned white. The tissue was placed in a new dish, and digested using type IV collagenase solution at 37°C for 20 min, followed by slight shaking (Hurwitz et al., 2019). The tissue supernatant was collected using the 70 μm nylon filter membranes.

Exosomes were extracted from the tissue supernatant by the method of ultracentrifugation. Firstly, the liver supernatant was centrifuged at 300 g (4°C) for 20 min to remove tissue fragments. Secondly, the supernatant was centrifuged at 2000 g (4°C) for 20 min to remove cell debris. Then the supernatant was centrifuged at 100,000 g (4°C) for 70 min to remove EVs with bigger diameters. Lastly, the supernatant was centrifuged at 100,000 g (4°C) for 70 min, and the exosome pellet was isolated and resuspended in 20 μl phosphate buffer saline (PBS, Biosharp, China). The last step was repeated twice. Each exosome extraction was performed from a single mouse liver and independently analyzed by mass-spectrometry.

### Western blot

Western blotting was performed according to a manufacturing procedure. The resuspended exosome samples were lysed with RIPA (Beyotime, China) with 1% PMSF (Beyotime, China), and the extracted proteins were separated using 12% SDS-PAGE gels (Beyotime, China) and then transferred onto 0.45 μm PVDF membranes (Millipore, United States). The membranes were blocked with 8% bovine serum albumin (BSA, BioFroxx, Germany) solution and incubated with primary antibodies (CD63 and TSG101, abcam, United States) in 8% BSA solution overnight at 4°C. After washing, the PVDF membranes were reacted with secondary antibodies for 1 h. At last, the blots were exposed using Genegenome XRQ Chemiluminescence system (Syngen, United Kingdom).

### Transmission electron microscopy

Exosome samples were fixed in 4% glutaraldehyde at 4°C overnight, followed by ultrathin sectioning. Images were captured and processed using a Hitachi-7500 transmission electron microscope (Hitachi Limited, Japan). The magnification of the electron microscope was 50,000×

### Extracellular vesicle size measurements by NanoSight

The exosome sample (10 µl) was diluted tenfold with PBS and inserted into a Nanosight instrument (NanoSight NS300, United Kingdom). Particles were tracked for 30s using Nanoparticle Tracking Analysis (NTA) V3.4 software.

### Tissue sample preparation and methyl chloroformate derivatization

The extracted metabolites were derivatized using a methyl chloroformate (MCF) approach based on the protocol published by Smart et al. (2010).

Treatment of Liver tissue: Firstly, equal quantities (30 mg) of tissue samples were weighed and obtained. Secondly, three magnetic beads, 200 µl of sodium hydroxide (1 M), 167 µl of methanol, and 20 µl of D4-alanine were added to each sample, followed by 30-second shock. Secondly, each sample was centrifuged at 2500 g (4°C) for 20 min, the supernatant was retrieved into another tube. Subsequently, 34 µl of pyridine and 20 µl MCF were pipetted, followed by 30 s of stirring, and another 20 µl of MCF followed by 30 s of stirring; Then 400 μl of chloroform and 400 μl of sodium bicarbonate (50 mM) were pipetted and vortexed for 10 s respectively. The lower chloroform phase was extracted for GC-MS analysis.

Treatment of exosome: Firstly, 100 μl resuspended sample, 100 μl sodium hydroxide (2 M) and 20 μl D4-alanine were mixed in a tube, followed by 30 min of heating in a 80°C metal bath. Secondly, 400 µl of methanol was added, followed by centrifugation at 2500 g (4°C) for 20 min, and the supernatant was retrieved into another tube. Subsequently, 34 µl of pyridine and 20 µl MCF were added, followed by 30 s of vortexing, and another 20 µl addition of MCF followed by 30 s of vortexing. Then 200 μl of chloroform and 400 μl of sodium bicarbonate (50 mM) were pipetted and stirring for 10 s respectively. The resulting lower chloroform phase was isolated for GC-MS analysis.

### Metabolite identification, data mining, and statistical analysis

The derivatized metabolites were analyzed using an Agilent Intuvo 9,000 linked to a MSD5977B with 70 eV of electron impact ionization. The gas capillary column was a BD-1701 (30 m × 250 μm id × 0.25 μm, Agilent). The derivatized samples were injected into a pulsed splitless mode inlet at 290°C with 1.0 ml/min flow rate of helium gas. The GC oven temperature was programmed from 45 to 180°C at rate 9.0°C, then to 220°C at rate 40.0°C, then to 240°C at rate 40.0°C, then to 280°C at rate 80°C. The temperatures of the auxiliary, MS quadrupole, MS source and guard chip were 250°C, 230°C, 150°C and 280°C, respectively. The mass range was detected from 30 to 550 µm. Scan speed was set to 1.562 µ/s and the solvent delay was applied until 5.5 min. Automated Mass Spectral Deconvolution & Identification System software was implemented for metabolite deconvolution. The metabolites compounds were identified by comparing the MS fragmentation patterns (mass-to-charge ratio and relative intensity of mass spectra to a reference ion) and respective GC retention time to an in-house MS library established using chemical standards. The remaining putative compounds were identified using a commercial NIST mass spectral library. The MassOmics R-based script was used to extract the relative concentration of the metabolites through the peak height of the most abundant fragmented ion mass. To improve quantitative robustness along with minimizing human and instrumental variability, the relative abundances of the identified compounds were normalized in the order of internal standards (D4-alanine), protein concentrations of exosomes measured using BCA Protein Assay Reagent (Beyotime, China), and liver tissue weight of the corresponding samples. Then, blank samples were used to subtract background contamination and any carryover from identified metabolites. Student’s *t*-test and false discovery rates (FDR) were performed in the R program to determine whether the concentration of each identified metabolite in exosomes or liver tissue was significantly different between PCOS and normal mice. Principle components analysis (PCA) was performed to compare exosomes and liver tissues metabolome profiles between different groups via Metaboanalyst 3.0 package for R (Smart et al., 2010). Adjusted logistic regression was performed to account for confounding factors and false discovery rates were reported to account for multiple comparisons. The area under the receiver operating characteristic (ROC) curve of the relative concentration of hair metabolites was calculated using the pROC R-package (Li et al., 2019), and multivariate ROC curves were constructed using a linear support vector machine model performed on the MetaboAnalyst website (https://www.metaboanalyst.ca). Metabolic pathway activity was estimated based on the Pathway Activity Profiling (PAPi) R-algorithm (Myers et al., 2004). The WGCNA analysis was performed on the BMKCloud website (www.biocloud.net). The metabolic network of each identified module of WGCNA was reconstructed based on the KEGG database through Cytoscape (Version 3.6.1).

## Results

### Features of Polycystic ovary syndrome and characters of exosomes

Basic information of each group was shown in Table 1. Compared to the normal group, the PCOS group showed increased numbers of cyst-like follicles and atretic follicles (*p* <0.05), as well as fewer normal follicles (*p* <0.05) (Figures 1B–D), which are the features of PCOS (Myers et al., 2004; Li et al., 2019; Qi et al., 2019). The exosomal marker proteins CD63 and TSG101 were visualized by western blotting (Figure 1E), the size of exosomes (50–100 nm) was determined by NTA (Figure 1G), and the bilayer-structure morphology of exosomes was identified by TEM (Figure 1G).

**TABLE 1:** Basic information of mice in each group.

Group	Young-age PCOS (YP)	Young-age normal (YN)	Middle-age PCOS (MP)	Middle-age normal (MN)
Number (*n*)	5	6	8	5
Age (week)	12 ± 2	12 ± 2	24 ± 2	24 ± 2
T (ng/ml)	3.53 ± 0.72	2.93 ± 0.25	3.07 ± 0.30	2.91 ± 0.20

### Overview changes of the metabolites

**FIGURE 2:**
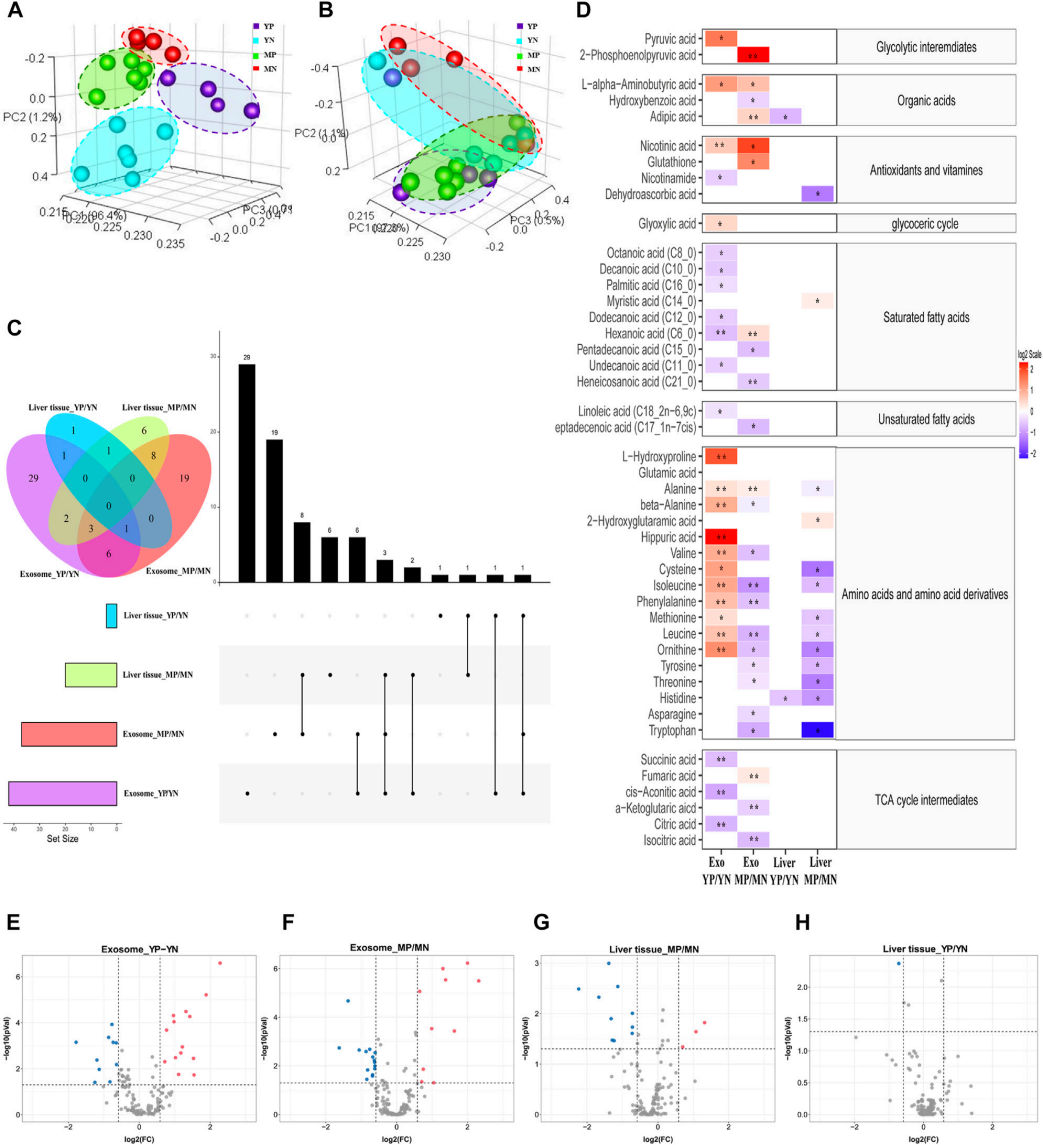
Principal component analysis (PCA) of metabolite patterns in **(A)** exosomes and **(B)** liver tissues. **(C)** Upset plot and Venn diagram of differential (*p* < 0.05) metabolites. The individual or connected dots represent the various intersections of metabolites that were either unique to or shared among comparisons. **(D)** Heatmap of the metabolites detected in each group showing the ratio of metabolites levels among the groups. Red color represents higher metabolites concentrations in PCOS groups than the normal groups, while blue color indicates lower levels in PCOS groups than the normal groups. The relative concentration of metabolites was plotted using a log2 scale, the significant fatty acids with p-values less than 0.05 are labelled with single asterisk, and q-values less than 0.05 are labelled with double asterisk. **(E–H)** Volcano plot of the differential (*p* < 0.05, FC > 1.5) metabolites in different positions. Red dots indicated upregulation, and blue dots indicated downregulation.

A total of 145 distinct metabolites were identified in liver tissues and exosomes using the in-house mass spectrometry library and NIST commercial database. Three-dimensional principal component analysis (3D-PCA) was performed, and the results showed that exosomes were clearly separated among four groups, while the liver tissue could not be accurately classified (Figures 2A,B). The multivariate analyses ANOVA and Tukey’s HSD test were applied to detect significant changes (*p* <0.05) in metabolites, and the numbers and intersections of analyses were visualized using Upset plot (Figure 2C). For the young-age group, 29 metabolites were significantly changed in exosomes comparing YP with YN mice, while one metabolite was significantly changed in liver tissue comparing YP with YN mice. For the middle age group, 19 metabolites were significantly changed in exosomes from MP mice compared to MN mice, while six metabolites were significantly changed in liver tissue from comparing MP with MN mice. A total of 48 metabolites in exosomes have significant differences without overlapping, nearly seven times more than those in liver tissue.

### Statistical analysis

To show the specifically increased and decreased production of metabolites, the volcano Plot and heatmap were plotted as in Figures 2D–H. In exosomes of YP mice, the quantities of alanine, valine, phenylalanine, leucine, isoleucine, L-alpha-aminobutyric acid, L-hydroxyproline, beta-alanine, L-ornithine and hippuric acid were significantly increased, while that of hexanoic acid, cis-aconitic acid, citric acid, and succinic acid were significantly decreased compared with that of exosomes from YN mice (FC> 1.5, *p* < 0.05, q < 0.05). In exosomes from MP mice, the quantities of fumaric acid, adipic acid, hexanoic acid and 2-phosphoenolpyruvic acid were significantly increased, while that of the phenylalanine, leucine, isoleucine, heneicosanoic acid,α-ketoglutaric acid and isocitric acid were significantly decreased compared with exosomes from MN mice (FC> 1.5, *p* < 0.05, q < 0.05). As for liver tissues, no metabolites showed a significant FDR (q < 0.05) difference between normal and PCOS groups for both young and middle age groups.

**FIGURE 3:**
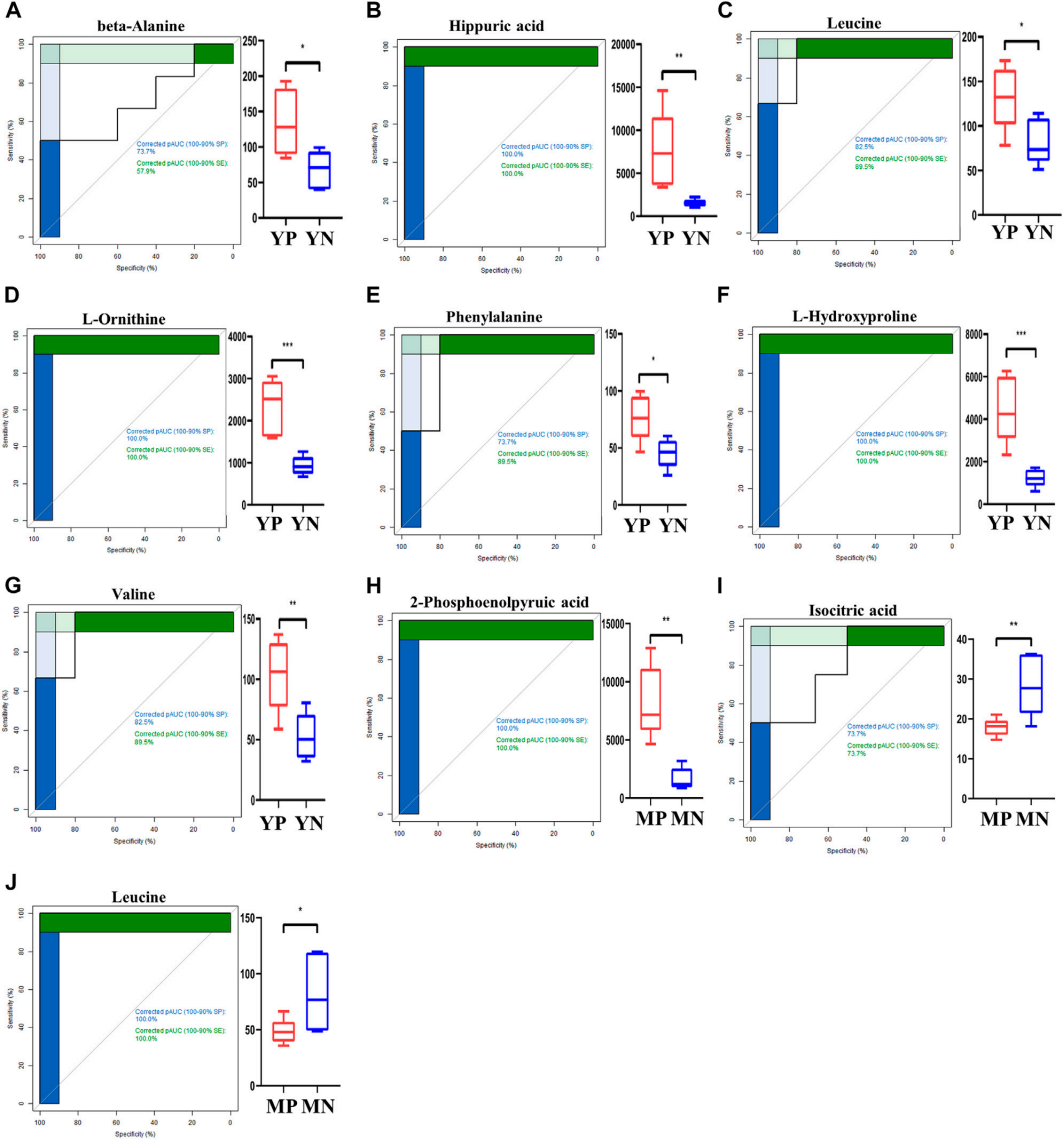
ROC curves for exosomal metabolites **(A–J)** with an area under the ROC curve above 90% (left panel). The levels of shortlisted metabolites are display as boxplots (right panel). Comparisons are either between young PCOS (YP) and young normal (YN), or middle age POCS (MP) and middle age normal (MN).

To further shortlist the identified metabolites, we performed the AUC (area under the ROC curve, AUC = 0.9) at the 95% CI (confidence interval) and plotted the ROC curves (Figure 3). At last, nine metabolites were identified as significantly differential metabolites (*p* < 0.05, q < 0.05, AUC >0.9). In exosomes of YP mice, the contents of alanine, hippuric acid, leucine, L-ornithine, phenylalanine, L-hydroxyproline, and valine were increased quantitatively. In exosomes of MP mice, the 2-phosphoenolpyruvic acid was upregulated, while the isocitric acid and leucine were decreased quantitatively.

### Metabolic pathway enrichment analysis

To understand the biological functions of metabolites identified, we annotated metabolic pathways using KEGG metabolic framework.

**FIGURE 4:**
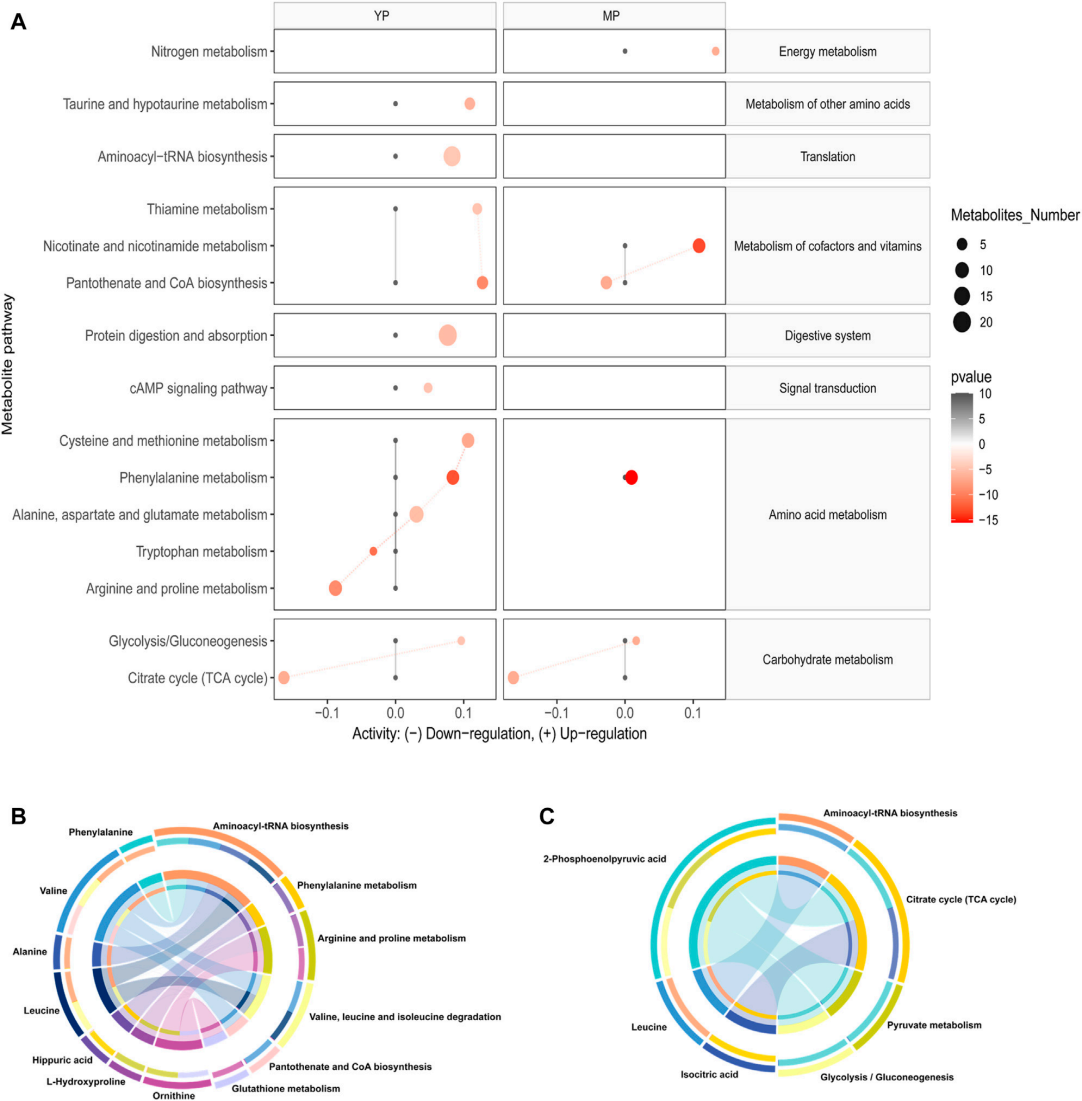
**(A)** Activities of metabolic pathways in liver-based on the exosomal metabolome of PCOS and normal mice at young and middle age. Black dots represent metabolic activities in exosome or liver tissue from normal mice that were adjusted to 0. Red dots represent metabolic activities in exosome or liver tissue from PCOS mice. The metabolic activities were visualized using the log_2_ scale. The dot size indicates the number of metabolites of the pathway, and the dot color indicates the p-value. Circos plots display the connectivity between metabolites and pathways at **(B)** young and **(C)** middle age.

Based on changes of exosomal metabolites, we performed the analysis of global metabolic pathways and the KEGG heatmaps were shown in Figure 4A. In YP liver, the taurine and hypotaurine metabolism, pantothenate and CoA biosynthesis, aminoacyl-tRNA biosynthesis, thiamine metabolism, protein digestion and absorption, cAMP signaling pathway, cysteine and methionine metabolism, phenylalanine metabolism, alanine, aspartate and glutamate metabolism, and glycolysis/gluconeogenesis were upregulated, while the tryptophan metabolism, arginine and proline metabolism and TCA cycle were downregulated. In MP liver, nitrogen metabolism, nicotinate and nicotinamide metabolism, phenylalanine metabolism, and glycolysis /gluconeogenesis were upregulated, while pantothenate and CoA biosynthesis and TCA cycle were downregulated.

Then we annotated the nine significantly differential (*p* < 0.05, q < 0.05, AUC > 0.9) metabolites, and the results showed that these metabolites were involved in the ten most relevant metabolic pathways: aminoacyl-tRNA biosynthesis; valine, leucine and isoleucine biosynthesis; phenylalanine metabolism, arginine and proline metabolism, valine, leucine and isoleucine degradation, pantothenate and CoA biosynthesis, glutathione metabolism, citrate cycle, pyruvate metabolism and glycolysis/gluconeogenesis (Figure 4B,C).

### Weighted gene co-expression network analysis

**FIGURE 5:**
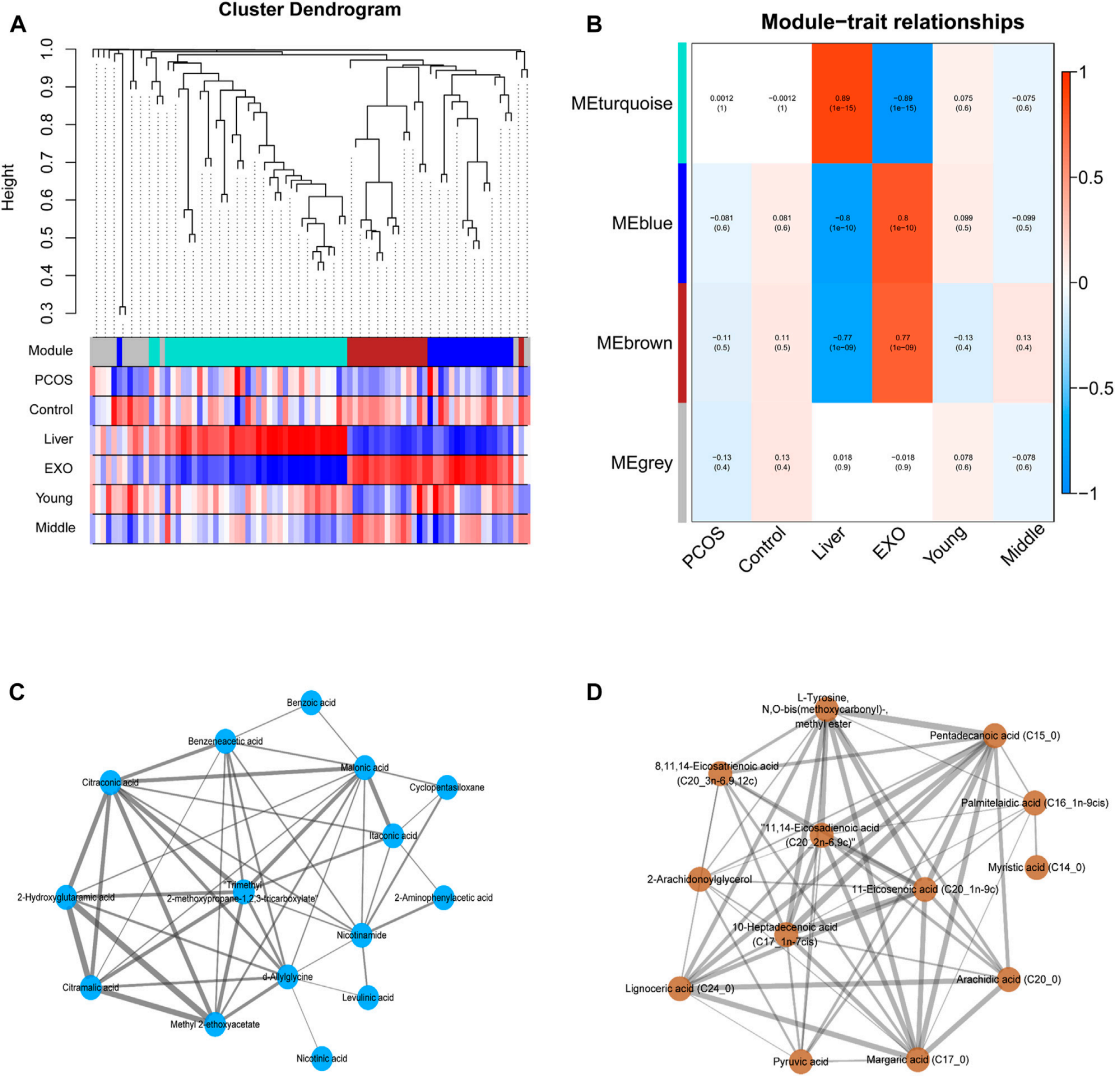
**(A)** The cluster dendrogram of co-expression network modules ordered by WGCNA based on the topological overlap matrix (TOM), each module was assigned different colors. **(B)** Heatmap of Module-trait relationships. Each row corresponds to a module eigengene (ME), and the column corresponds to a trait. Each cell contains the corresponding correlation and p-value. **(C–D)** The metabolites and their correlation in each ME.

WGCNA was performed using the Pearson’s correlation matrix, and the hierarchical clustering dendrograms of identified co-expressed metabolites was shown in Figure 5A. Three key modules (brown, turquoise, and blue) were identified, and the grey module represented the group of metabolites that had outlying correlation with others. The association between traits and module epigenegens (MEs) were performed, and the results were shown using the heatmap (Figure 5B). The main members and their associations were shown using network diagrams (Figures 5C,D). In particular, most of the metabolites within the brown module were fatty acids, including five saturated long-chain fatty acids and five unsaturated long-chain fatty acids. Both the blue and turquoise modules appeared to have a high correlation with “exosome”, suggesting that the metabolites falling in these modules were mainly enriched in exosomes rather than expressed in cells of liver tissues.

## Discussion

**FIGURE 6:**
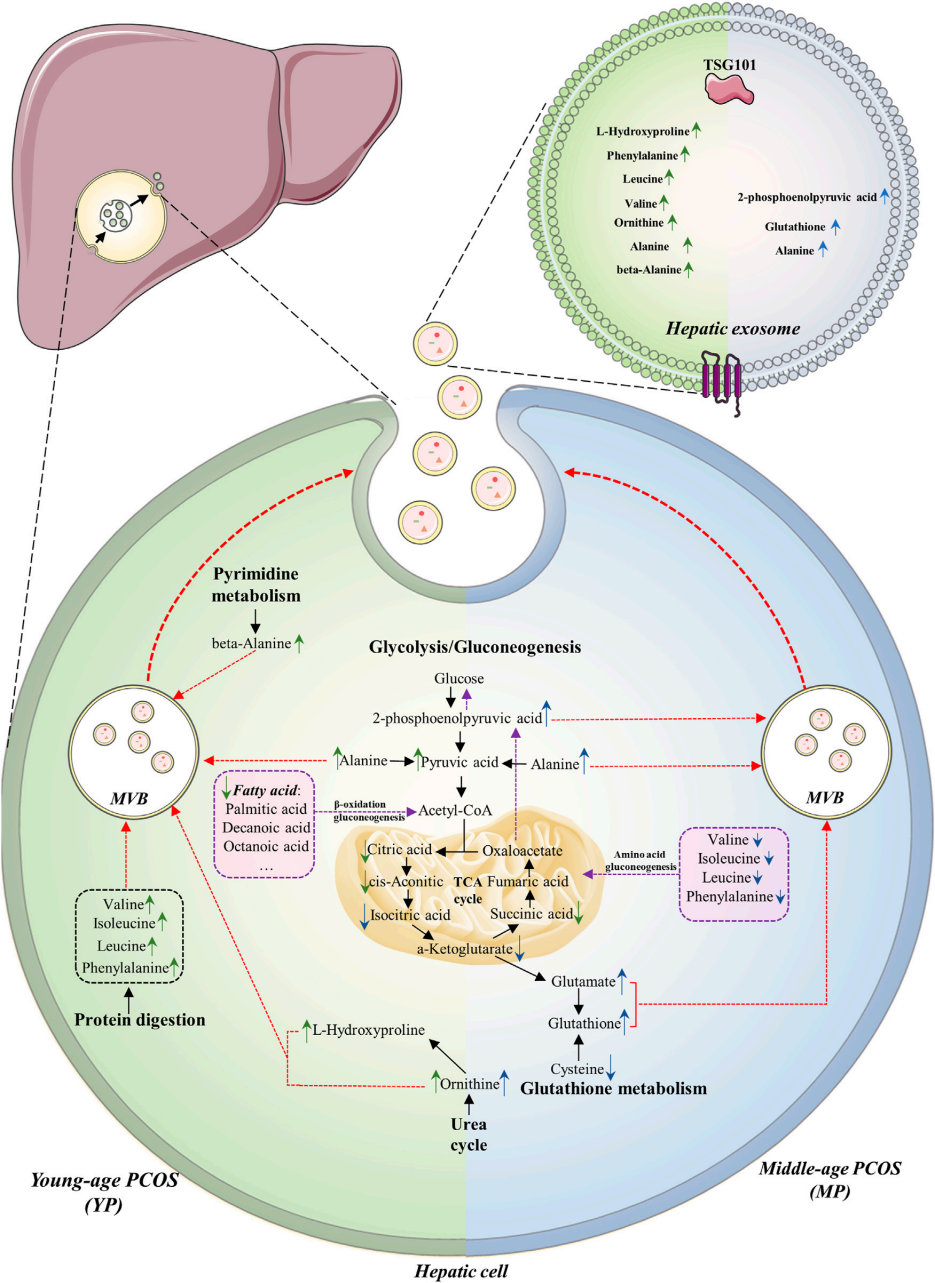
Summary of altered metabolites and metabolic pathways in this study. The metabolome of liver tissue and hepatic exosomes in PCOS between young (YP, left green part) and middle age (MP, blue right part) are compared. The common alterations of the two age groups are downregulation of glycolysis and TCA cycle, as well as upregulation of gluconeogenesis. The major substrates for gluconeogenesis in YP and MP hepatic cells are fatty acids and amino acids, respectively (purple boxes). In YP hepatic cells, the urea cycle, protein digestion and pyrimidine metabolism seem to be upregulated, while the glutathione metabolism seems to be upregulated in MP hepatic cells. The arrows indicate up- or down-regulation of metabolites in (green) young- or (blue) middle-age groups detected in the liver or exosomes. The purple dotted lines indicate the gluconeogenesis pathway in hepatic cells. The red dotted lines indicate the processes in which the elevated metabolic products are transported into exosomes.

As a common metabolic disorder of women, PCOS can affect many organs including the liver. Exosomes are lipid-bilayer-structure microparticles secreted by almost all cell types and the contents of exosomes can be used to reflect the pathological and physiological states of parental cells or source organs. In this study, we investigated the metabolic changes of liver tissue and hepatic exosomes from the young- and middle-age PCOS mice and compared them with those in the control health mice. The changes of metabolite profiles in liver exosomes were more obvious than those in liver tissues, suggesting a stronger response of exosomes when facing pathophysiological changes. Our findings clearly demonstrated that PCOS is associated with aberration in glycolysis and TCA cycle. There was also a shift of substrate preference from fatty acids to amino acids for gluconeogenesis when PCOS mice aging (Figure 6).

### Exosomal metabolome can reflect the liver metabolic status

In order to explore the metabolic dialogues between hepatic cells and their exosomes, it is necessary to understand how exosomes mediate hepatic physiology. The metabolic states of the cells influence the biological markers of exosomes, which can prospectively reflect the state of the parental cell. The statistical analysis results showed more significant changes in metabolite profiles inside the exosomes than those in the liver tissue. PCA of the global metabolic profiles revealed that liver tissue was closely clustered together and hard to be separated, while the exosomes were easily separated between different groups. Moreover, the differential metabolites in the exosome were nearly sevenfold higher than those in liver tissue (Figure 2). These results showed more prominent changes in exosomes compared to those in liver tissue, indicating that exosomes might respond more actively towards metabolic disruption than liver tissue. Wang *et al* reported that the change of miR-21 in the exosome of hepatocellular carcinoma (HCC) patients was more prominent than that in whole blood, a supporting evidence of our view (Wang et al., 2014; Wang et al., 2019). This unique property of exosomes is likely to be contributed by the biogenesis and the structure of exosomes. One of the most currently recognized mechanisms for biogenesis is the double invagination of intracellular multivesicular bodies (MVBs) (Kowal et al., 2014; Zhang et al., 2019; Kalluri and LeBleu, 2020). Because of the double invaginations, various cell-specific components from the cell membrane or cytoplasm were incorporated into the exosome. Furthermore, the special double-layered membrane structure of exosomes could protect their contents from degradation by various enzymes (Shao et al., 2018; Hu et al., 2020), which could be used to explain why some metabolites in this study were significantly elevated in exosomes compared to those in the liver. Since several studies suggested that exosomes can be used to screen for disease and even predict the stage of disease development (Hoshino et al., 2015; Puhka et al., 2017; Hoshino et al., 2020), exosome metabolome may be conducive to the comprehensive study of hepatic pathophysiology and worth being further investigated.

### The common metabolic changes are likely to be associated with Polycystic ovary syndrome in both age groups

To elucidate the underlying metabolic alterations associated with PCOS, shared metabolic changes in both age groups were discovered and analyzed. We found that aberrant glucose metabolism was related to PCOS and was independent of age. Higher levels of glycolysis/gluconeogenesis intermediates, including 2-phosphoenolpyruvic acid and pyruvic acid were detected in PCOS exosomes in both age groups. Meanwhile, reduced concentrations of TCA cycle intermediates were observed in the PCOS liver across age groups (Figure 2). Impaired glucose metabolism is an important hallmark of PCOS, but due to the different types of specimens used in individual study, the results and conclusions remained controversial, no consensus was achieved so far. Sun et al. (2012) and Zhao et al. (2012) reported the increased glycolysis and decreased TCA cycle in their plasma metabolomic analysis in women with PCOS. Zou et al. (2018) reported that some TCA cycle intermediates, including α-ketoglutarate and cis-aconitate, were upregulated in PCOS using urine samples. Zou et al. (2018) reported that the TCA cycle was upregulated in the microenvironment of follicles and oocyte development in PCOS using follicular fluid samples. In this study, however, the alteration of glucose metabolism was somehow different. Insulin resistance (IR) is a common feature of PCOS, which compromises the hepatic sensitivity to insulin, inhibits its glucose catabolism, and makes insulin unable to effectively suppress hepatic gluconeogenesis (Wei et al., 2018). Glycolysis and TCA cycle are important steps in glucose metabolism which might be downregulated due to IR, and the increased 2-phosphoenolpyruvic acid was mainly produced by the process of gluconeogenesis. In alignment with this assumption, the significant increase of alanine in PCOS exosomes in both ages (Figure 2) might be another evidence of upregulated gluconeogenesis. Alanine is an essential part of the glucose-alanine cycle in gluconeogenesis. In some physiological states, the alanine could be transported to the liver through the glucose-alanine cycle to generate pyruvate, which is a source of carbon for producing glucose (Felig et al., 1970). Therefore, our results indicated upregulation of gluconeogenesis as well as downregulation of glycolysis and TCA cycle in the PCOS liver.

### The differences of metabolic states between Polycystic ovary syndrome mice at young and middle age

Since PCOS is an endocrine disease associated with hormones, age is an important modifying factor which should be taken into consideration when doing analysis. Livadas et al. (2014) reported that aging could increase IR in obese women with PCOS; they suggested that PCOS represented a moving fluctuation of hormonal to metabolic abnormalities, as women with the PCOS were aging. The effects of the age factor on PCOS exosomal metabolome was also highlighted in this study. Our results showed that metabolite profiles in exosomes exhibited different trends across different age groups. Although hepatic gluconeogenesis was upregulated in both age groups, different carbon sources were utilized in each group. In YP groups, the fatty acids were utilized for gluconeogenesis through β-oxidation, causing the reduction of fatty acids in exosomes. Moreover, it had been reported that a complete set of fatty acids were loaded into exosomes during biogenesis in the parental cell (Record et al., 2014), and the component of the brown module identified by WGCNA analysis also illustrated that the hepatic exosomes might have specific enrichment of some fatty acids (Figure 5). Record et al. (2014) reported that exosomes convey not only bioactive lipids but also transport lipolytic enzymes. Thus, another possible reason for the decrease of fatty acids is the degradation by enzymes carried in exosomes. In MP groups, the amino acids including valine, isoleucine, leucine, phenylalanine were utilized as substrates for gluconeogenesis, which led to decreased amino acids in both MP exosomes and liver tissues. Since IR has a high prevalence and strong association with many metabolic disorders, it is often considered to have important roles in many adverse aging phenotypes and age-related conditions (Barzilai and Ferrucci, 2012). Thus, we speculated that ageing could worsen the IR, which can probably lead to a decrease in the ability of hepatic fatty acid metabolism, and then make the precursor of gluconeogenesis shift from fatty acids to amino acids. Understanding the differences in substrate utilization between young- and mid-age PCOS mice may provide a potential adjuvant therapeutic intervention for PCOS at different ages.

In addition to gluconeogenesis, some unique metabolic characteristics were found in the PCOS liver at different ages. In YP exosomes, significantly higher levels of exosomal amino acids, including valine, isoleucine, leucine and phenylalanine, were detected, which was contrary to the observed trends in MP exosomes. Valine, isoleucine, and leucine are branched-chain amino acids (BCAA), which are essential amino acids associated with positive effects on glucose homeostasis (Lynch and Adams, 2014). Several studies indicated that the BCAA contributed to the development of obesity-associated insulin resistance (IR) (Newgard et al., 2009; Rajska et al., 2020). Phenylalanine is an aromatic amino acid (AAA) and is also proved to be associated with IR (Würtz et al., 2013). Since BCAA and phenylalanine are essential amino acids, they can only be uptaken from a dietary source. Together with the result of pathway analysis (Figure 4A), the increased levels of these essential amino acids might be evidence of enhanced protein catabolism in the YP liver. Furthermore, ornithine is a non-essential amino acid and is a part of the urea cycle. Kyselova et al. (2019) reported a higher level of ornithine in PCOS plasma, and the author then proposed that ornithine level could be an early biomarker of potential cardiovascular disease in PCOS patients. β-Alanine is one of the nucleotide decomposition products, which is mainly produced by cytosine decomposition in the cytoplasm. The level of β-alanine level in the YP exosome was specifically elevated, indicating that pyrimidine catabolism activity of hepatic cells in YP mice may be impaired. Moreover, the pyrimidine metabolism pathway has also been reported to play a role in the risk of PCOS (Wei et al., 2018). On the other hand, the glutathione (GSH) was significantly increased in MP exosomes. GSH is an antioxidant involved in protecting cells from oxidative damage, and it has been reported that oxidative stress is a key mechanism of developing IR (Diane et al., 2020) and the pathophysiology of PCOS. Thus, we speculated that compared to YP groups, the increased age imposed greater stress on MP liver, leading to the level increase of GSH, thereby providing increased antioxidant defense to protect themselves from toxic reactive oxygen species (ROS) (Mohamed et al., 2020). Together, we thought that the different metabolic characterizations between YP and MP groups could be attributed to insulin resistance, which was also influenced by aging.

### Limitation

Despite the promising results, several limitations of our research also merit discussion. Firstly, the downstream evaluation of metabolomic results should be established. These include knocking-down genes or inhibiting enzymatic activities involved in the TCA cycle, gluconeogenesis, GSH metabolism, and protein digestion in the mouse liver with PCOS. Secondly, further studies investigating the effects of PCOS hepatic exosomes on the liver cells *in vitro* are warranted to verify the metabolic roles of exosomes in PCOS pathogenesis. Lastly, in order to comprehensively understand the metabolic status of liver tissue and hepatic exosomes in PCOS, multi-omics approaches should be integrated into the subsequent research.

## Conclusion

This is the first metabolomics research of hepatic exosomes in PCOS. Our findings indicated that the metabolite profile in the exosome has the potential to reflect the metabolic alterations of the liver. The downregulations of glycolysis and TCA cycle seem to be associated with hepatic pathophysiology of PCOS independent of age. Besides, substrate utilization of gluconeogenesis differs between YP and MP liver and it highlights the influence of aging on PCOS progression.

## Data Availability

The original contributions presented in the study are included in the article/Supplementary Materials, further inquiries can be directed to the corresponding authors.

## Abbreviations

PCOS, Polycystic ovary syndrome; IR, Insulin resistance; GC-MS, gas chromatograph-mass spectrometry; YP mice, young-age PCOS mice; YN mice, young-age normal mice; MP mice, middle-age PCOS mice; MN, middle age normal mice.
