# Supplementary material for: The complex metabolic interactions of liver tissue and hepatic exosome in PCOS mice at young and middle age
Source: Front Physiol. 2022 Sep 20;13:990987. doi: 10.3389/fphys.2022.990987 (PMC9531160; doi:10.3389/fphys.2022.990987)
Supplement: Supplementary file 1 [file Table1.DOCX]

**Supplementary Table 1. List of metabolites in each sub-group for the Upset plot**

| **Sub-group** | **Number** | **Metabolites** |
| --- | --- | --- |
| Liver_Old-PCOS/Old-Control | 6 | 2-Hydroxyglutaramic acid, alanine, cabamic acid, creatinine, dehydroascorbic acid, myristic acid (C14_0) |
| Liver_Young-PCOS/Young-Control | 1 | Azetidine |
| Liver_Young-PCOS/Young-Control,  Liver_Old-PCOS/Old-Control | 1 | Histidine |
| EXO_Old-PCOS/Old-Control | 19 | (E,S)-2-Hexenoic acid, 10-heptadecenoic acid (C17_1n-7cis), propanoic acid, asparagine, benzoic acid, 4-methoxy-ethyl ester-Dimethyl (S)-(-)-malate, O-methoxycarbonyl-DL-3-aminoisobutyric acid, fumaric acid, glutamic acid, glutaric acid, glutathione, heneicosanoic acid (C21_0), hydroxybenzoic acid, isocitric acid, N-acetylglutamic acid, nicotinic acid, pentadecanoic acid (C15_0), sarcosine, tricarboxylate |
| EXO_Old-PCOS/Old-Control,  Liver_Old-PCOS/Old-Control | 8 | 2-Phosphoenolpyruvic acid, fumaric acid (10.49min), L-tyrosine, Methyl-1-acetylpiperidine-2-carboxylate, ornithine, threonine, tryptophan, tyrosine |
| EXO_Young-PCOS/Young-Control | 29 | 7,9-Di-tert-butyl-1-oxaspiro(4,5)deca-6,9-diene-2,8-dione, acetamide, 2-(1-benzimidazolyl)-azelaic acid, beta-alanine, cis-aconitic acid, citric acid (18.37min_main), cyclotetrasiloxane, octamethyl-d-allylglycine, decanoic acid (C10_0), Dimethylaminomalonate hydrochloride, dodecane, dodecanoic acid (C12_0), glyoxylic acid, hexanoic acid (C6_0), hippuric acid, itaconic acid, L-hydroxyproline, linoleic acid (C18_2n-6,9c), L-leucine, N-acetyl-methyl ester-L-ornithine, N,N'-bis(methoxycarbonyl)- methyl ester- nicotinamide, octanoic acid (C8_0), palmitic acid (C16_0), p-xylene, pyruvic acid, succinic acid, tetrachlorethane, trans-2-Methyl-2-butenedioic acid dimethylester, undecanoic acid (C11_0) |
| EXO_Young-PCOS/Young-Control,  Liver_Old-PCOS/Old-Control | 2 | Methionine, cysteine |
| EXO_Young-PCOS/Young-Control,  Liver_Young-PCOS/Young-Control | 1 | d-Allylglycine-N-ethoxycarbonyl |
| EXO_Young-PCOS/Young-Control,  EXO_Old-PCOS/Old-Control | 6 | 2-Arachidonoylglycerol, a-ketoglutaric aicd, alanine, L-alpha-aminobutyric acid, phenylalanine, valine |
| EXO_Young-PCOS/Young-Control, EXO_Old-PCOS/Old-Control,  Liver_Old-PCOS/Old-Control | 3 | 2-Aminophenylacetic acid, isoleucine, leucine |
